# Selective Inhibition of HDAC1 by Macrocyclic Polypeptide for the Treatment of Glioblastoma: A Binding Mechanistic Analysis Based on Molecular Dynamics

**DOI:** 10.3389/fmolb.2020.00041

**Published:** 2020-03-11

**Authors:** Yang Zhang, Tingting Fu, Yuxiang Ren, Fengcheng Li, Guoxun Zheng, Jiajun Hong, Xiaojun Yao, Weiwei Xue, Feng Zhu

**Affiliations:** ^1^College of Pharmaceutical Sciences, Zhejiang University, Hangzhou, China; ^2^School of Pharmaceutical Sciences, Chongqing University, Chongqing, China; ^3^State Key Laboratory of Applied Organic Chemistry and Department of Chemistry, Lanzhou University, Lanzhou, China

**Keywords:** HDAC, macrocyclic peptides, molecular docking, MD simulation, binding free energies, interaction fingerprints

## Abstract

Glioblastoma (GBM) is the most common and aggressive intracranial malignant brain tumor, and the abnormal expression of HDAC1 is closely correlated to the progression, recurrence and metastasis of GBM cells, making selective inhibition of HDAC1 a promising strategy for GBM treatments. Among all available selective HDAC1 inhibitors, the macrocyclic peptides have gained great attention due to their remarkable inhibitory selectivity on HDAC1. However, the binding mechanism underlying this selectivity is still elusive, which increases the difficulty of designing and synthesizing the macrocyclic peptide-based anti-GBM drug. Herein, multiple computational approaches were employed to explore the binding behaviors of a typical macrocyclic peptide FK228 in both HDAC1 and HDAC6. Starting from the docking conformations of FK228 in the binding pockets of HDAC1&6, relatively long MD simulation (500 ns) shown that the hydrophobic interaction and hydrogen bonding of E91 and D92 in the Loop2 of HDAC1 with the Cap had a certain traction effect on FK228, and the sub-pocket formed by Loop1 and Loop2 in HDAC1 could better accommodate the Cap group, which had a positive effect on maintaining the active conformation of FK228. While the weakening of the interactions between FK228 and the residues in the Loop2 of HDAC6 during the MD simulation led to the large deflection of FK228 in the binding site, which also resulted in the decrease in the interactions between the Linker region of FK228 and the previously identified key amino acids (H134, F143, H174, and F203). Therefore, the residues located in Loop1 and Loop2 contributed in maintaining the active conformation of FK228, which would provide valuable hints for the discovery and design of novel macrocyclic polypeptide HDAC inhibitors.

## Introduction

Glioblastoma (GBM) is the most common and aggressive intracranial malignant brain tumor with the median survival duration <2 years in spite of chemotherapy, radiation or surgical resection (Natsume et al., 2019). In the current chemotherapies, such as *temozolomide*, drug resistance is the predominant obstacle (Zhang et al., 2012; Chen et al., 2019; Kim et al., 2019; Rahman et al., 2019; Su et al., 2019; Xingyi et al., 2019). On the basis of the latest experimental results obtained from the large-scale profiling which included the whole exome and RNA sequencing, it can be learnt that genetic and epigenetic mechanisms are involved in the occurrence and progress of glioma cells (Cancer Genome Atlas Research, 2008; Brennan et al., 2013), especially the aberrant epigenetic silencing of genes caused by histone deacetylation (Vaissiere et al., 2008; Cartron et al., 2013). A large number of researches have proven that significant nuclear expression of histone deacetylase 1 (HDAC1) occurred in GBM cells during the process of tumor progression, recurrence, and metastasis (Bhat et al., 2008; Kim et al., 2008; Campos et al., 2011; Li et al., 2016, 2018a; Zhang et al., 2016; Staberg et al., 2017; He et al., 2019; Natsume et al., 2019). In addition, the invasive and proliferative phenotype of GBM cells was found to be related to the overexpression of HDAC1 level (Han et al., 2013). Moreover, HDAC1 inhibitors developed for a variety of tumors have been extensively tested in clinical trials as a single drug or in combination with other chemotherapy agents (Lu et al., 2008; Tan et al., 2010; Campos et al., 2011; Dong et al., 2013; Tang et al., 2018). Currently, four HDAC inhibitors (HDACi) including Vorinostat, Romidepsin, Panobinostat, and Belinostat have been approved by FDA for anticancer therapeutics, and some other HDAC inhibitors (such as Ricolinostat) are still in the clinical trials to treat hematological and solid malignancies (Yang et al., 2016; Eckschlager et al., 2017; Li et al., 2018b).

**Figure 1:**
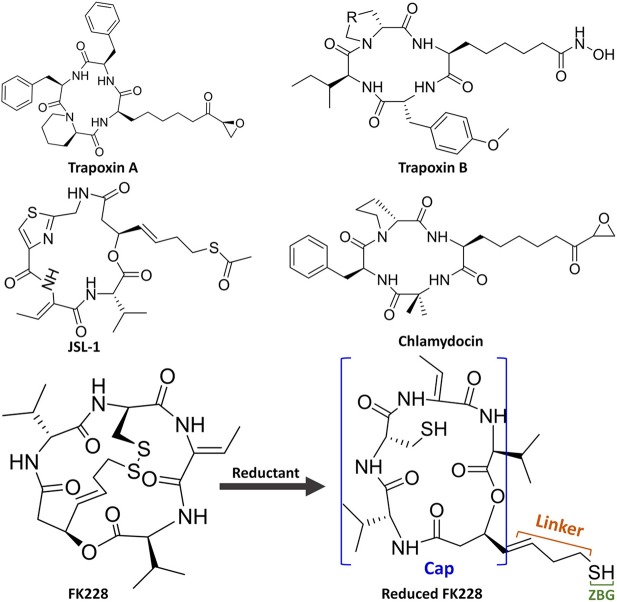
Molecular skeletons of HDAC inhibitors with macrocyclic Cap group.

Unfortunately, there are no clinical or approved cases of HDACi currently effective for the treatment of GBM. This is because targeting the key epigenetic enzymes, oncogenes, and pathways specific to glioblastoma cells by the drugs has proved to be of great challenges (Sturm et al., 2014), for example, the lower effective inhibitory concentrations within the tumor cells and adverse toxicological effects (Lee et al., 2015). In order to overcome the shortcoming caused by the limited stability and unacceptable pharmacokinetic properties of most existing drugs or molecules, various molecular skeletons were designed to improve the HDAC1-based drugs development, which conform the pharmacophore model of traditional HDACi, namely containing Cap group (Cap), Connect unit (CU), Linker region (Linker), and Zinc Binding Group (ZBG) (Figure 1; Dehmel et al., 2008; Varasi et al., 2011; Choi et al., 2012; Giannini et al., 2014; Krieger et al., 2017). Among these pharmacophores, the ZBG should penetrate deep into the bottom of the active pocket and chelate with zinc ion located in the catalytic center to compete with the protein for zinc ion, thereby inhibiting the catalytic activities of HDACs. And such binding pattern in the active pocket is the active conformation of the HDAC inhibitors (Krieger et al., 2019; Shen et al., 2019; Vergani et al., 2019).

Interestingly, the skeletons with macrocyclic Cap have better inhibitory activities against HDAC Class I than Class II, among which the macrocyclic peptide inhibitors account for a large proportion (Mwakwari et al., 2010; Rajak et al., 2013; Tapadar et al., 2015). As inhibiting class II HDACs (represented by HDAC6) can lead to unwanted toxic and side effects (especially serious cardiac toxicity) (Roche and Bertrand, 2016), targeting specific HDAC subtypes has shown great therapeutic potential. The macrocyclic HDACi targeting only Class I HDAC family or HDAC1 are regarded as lower toxicity and more tolerable than pan-HDAC inhibitors, which have shown great potential values of clinical therapeutic effects (Benelkebir et al., 2011; Bhansali et al., 2011; Mallinson and Collins, 2012; Salvador et al., 2014; Decroos et al., 2015; Pilon et al., 2015; Chen et al., 2017; Cheng et al., 2017; Kim et al., 2017). However, there are currently no crystal structures of HDAC1 and HDAC6 complexed with macrocyclic HDACi that have been resolved. Therefore, it is urgent and necessary to reveal the difference in the binding mechanism of macrocyclic HDACi in HDAC1&6 at the atomic level.

In this study, Romidepsin (FK228) was applied as a case study to investigate why macrocyclic polypeptide inhibitors tend to inhibit HDAC1, and various computational approaches were adopted to explore the binding modes of FK228 in HDAC1&6. First, the studied complexes of FK228 in HDAC1&6 were constructed via molecular docking approach. Second, the docked results were further verified by molecular dynamic (MD) simulation. Finally, the key residues responsible for the difference in binding energy of macrocyclic HDACi in HDAC1&6 were identified. In summary, the mechanism underlying why FK228 prefer to inhibit HDAC1 was elaborate through differential energy contributions and interaction fingerprints among the identified key amino acids, which could provide valuable information for the drug discovery on the basis of selective inhibition of HDAC1 in the future.

## Results and Discussion

### Construction of FK228 Complexed HDAC1and6 Structures

**Figure 2:**
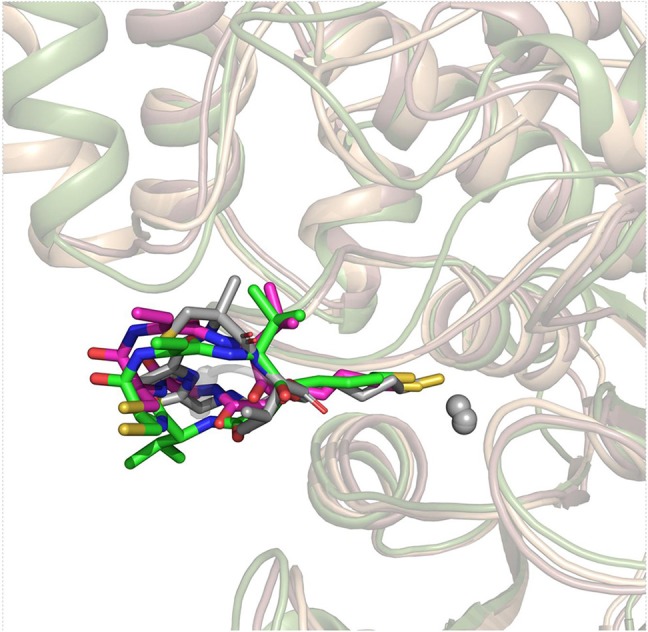
Superimposition of FK228 in HDAC1&6 and HDAC8 complexed with depsipeptide inhibitor.

On the basis of the resolved protein crystals of HDAC1&6 available in Protein Data Bank (PDB) (Hai and Christianson, 2016; Watson et al., 2016), there were 75 and 68 binding poses of FK228 in HDAC1 and HDAC6 generated by molecular docking, respectively. Except for the docking score, the spatial similarity of the docking pose to the largazole thiol in HDAC8 was considered in selecting the conformations of FK228 in HDAC1&6 (Figure 2). This was because there were no HDAC1&6 crystals resolved with macrocyclic inhibitors, and HDAC8 (HDAC Class I) protein crystal with similar active pocket as HDAC1&6 complexed with largazole thiol could provide important clues for the choice of the initial conformations of FK228 in HDAC1&6 (Figure 2). According to Figure S1, it could be learnt that binding sites of HDAC1&6 were mainly composed of loop regions, namely loop 1–7. In addition, the selected docked poses suggested that the Cap group of FK228 had interactions with the residues at the rim of the active pocket of HDAC1&6, and the Linker coupled with ZBG penetrate the active pocket, which made the ZBG chelating with the zinc ion in catalytic center. Moreover, the orientation of FK228 in HDAC1&6 is highly coincident with the largazole thiol inhibitor in HDAC8 (Figure 2), which verified the reliability of the docking conformation to some extent. According to the Tables S1, S2, it could be found that the RMSD values were basically negatively correlated with the absolute value of the docking scores, and the smaller RMSD values could reflect the better binding of FK228 in the HDAC1&6 to some extent. In order to verify the reliability of the experiments, one additional initial conformation of the constructed system have been selected for the further molecular dynamic simulation (Figure S2).

### Evaluating the Stability of MD Simulation via RMSD Analysis

#### The Complexes Stabilities Along the Simulation Monitored by RMSD

**Figure 3:**
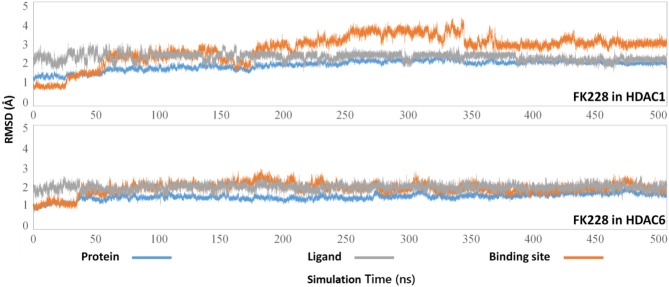
Root mean square deviations of protein backbone atom, ligand heavy atoms, and the backbone atoms of the residues in the binding site as the function of time in MD simulations.

The selected docking conformations of HDAC1&6 in complex with FK228 were sampled by 500 ns MD simulation, and the dynamic trajectories of the studied complexes were supervised through the RMSD plots of the backbone-atoms of HDAC1&6, heavy-atoms of FK228, and the backbone-atoms of the amino acids in the binding pocket (within 5 Å of ligand) as the function of simulation time (Figure 3). Insight from the RMSD values in Figure 3, the FK228-HDAC1 and FK228-HDAC6 systems reached the equilibrium states around 350 and 50 ns, respectively. Moreover, according to the RMSD values of the additional independent simulations also showed that the constructed systems reached the equilibrium around 200 ns (Figure S3), and the difference in the fluctuation of the binding site of the two simulations were caused by the flexible loop domain.

#### The Conformational Rearrangements of FK228 in HDAC1and6

**Figure 4:**
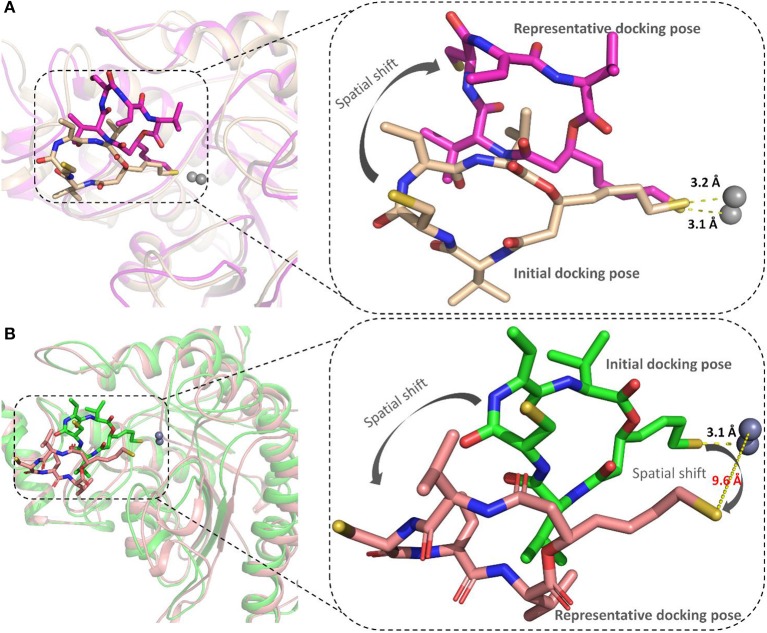
Comparison of the initial conformation and the representative conformation of the FK228 in HDAC1&6: **(A)** FK228 in HDAC1 system; **(B)** FK228 in HDAC6 system.

The representative structures of FK228 binding to HDAC1&6 were obtained from the equilibrated trajectories and were compared with their corresponding initial conformations (Figure 4). During the MD process, the protein conformational changes were calculated by *VMD* software, the values were 1.85 and 1.92 Å for the HDAC1-FK228 and HDAC6-FK228 systems, indicating the small change in the conformation of protein. According to Figure 4A, it can be learnt that slight spatial shift of FK228 occurred in HDAC1 active site and the binding conformation maintain the interaction of sulfhydryl group (ZBG) chelating with the zinc ion (~3.2 Å) through inserting deeply into the active pocket. In contrast, for FK228 in HDAC6 (Figure 4B), there was a large deflection of the ZBG in the ligand from the initial conformation, namely straying from the catalytic center (~9.6 Å). In order to verify the reliability of the experiment, the conformational rearrangement of FK228 in HDAC1&6 of the additional independent experiment was also analyzed, and based on Figure S4, it could learnt that FK228 could maintain the active conformation in HDAC1 but not in HDAC6 (ZBG was also far away from the zinc ion). The conformational rearrangements investigated by MD simulation imply that the protein-ligand binding modes is the leading cause of the significant difference of FK228 inhibitory activity to HDAC1&6 and need to be further explored.

### Molecular Mechanism of FK228 Selectivity to HDAC1and6

#### Insights From the FK228-HDAC1and6 Interaction Fingerprints

**Figure 5:**
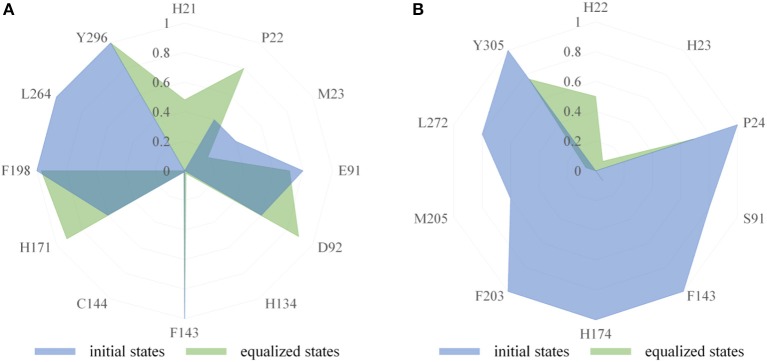
Comparison of interaction fingerprints of FK228 in HDAC1&6 in the final 50 ns simulations with that of the optimized docking poses: **(A)** interaction fingerprints of FK228 in HDAC1; **(B)** interaction fingerprints of FK228 in HDAC6.

The binding modes of FK228 in HDAC1&6 are related to the interactions between drugs and amino acids of the target proteins. Thus, the interaction fingerprints analysis was used to explore the difference of FK228-HDAC1&6 binding modes (Figure 5). Figure 5A indicates that FK228 can maintain its interactions with the P22, E91, and D92 located at Loop1 and Loop2 of HDAC1 before and after MD simulation. For HDAC6-FK228 complex, although FK228 can maintain the interaction with P24 of Loop1, the interaction with S91 of Loop2 in the initial conformation was disappeared after MD simulation (Figure 5B). In addition, according to the interaction fingerprints, E91 locating on the Loop2 of HDAC1 contributed to a strong hydrophobic interaction with FK228, and the corresponding site on HDAC6 has no interaction with FK228, leading to the weak interaction between FK228 and Loop2 of HDAC6, which is the main reason of the large spatial shift of FK228 in the binding site of HDAC6.

#### Insights From the Calculated Binding Free Energy of FK228-HDAC1and6 Complexes

**Figure 6:**
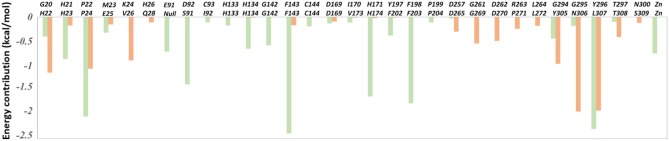
The per-residue binding free energy decomposition of 31 residues with high energy contribution (≥0.1 kcal/mol) to the interaction in at least one studied complex: FK228 in HDAC1 (light green); FK228 in HDAC6 (light orange).

The total binding free energies of HDAC1-FK228 and HDAC6-FK228 were −37.01 and −27.84 kcal/mol, which was consistent with the inhibitory gradient of FK228 toward HDAC1 and HDAC6 (Table 1). To qualify the energy contribution of each amino acid in HDAC1&6 for FK228's binding, the total binding free energies were decomposed at amino acid basis and the important ones with high contribution (≥0.1 kcal/mol) (Zheng et al., 2017) were identified. As shown in Figure 6 the values of amino acids energy with high contribution in each complex varied significantly (taking FK228-HDAC1 as example, the contribution of F143 equaled to −2.39 kcal/mol, which was almost 22 times of C93's energy contribution). As expected, the contributions of the amino acids at the corresponding position on HDAC1&6 also varied greatly. Taking G295 in HDAC1 and N306 in HDAC6 as example, it contributed −0.18 and −1.94 kcal/mol to the binding of FK228 in HDAC1 and HDAC6, respectively.

**Table 1:** Calculated and experimental data of FK228 binding to HDAC1 and HDAC6 (ΔG is in kcal/mol and IC50 value is in nM).

**Systems**	**ΔE_**ele**_**	**ΔE_**vdW**_**	**ΔG_**pol**_**	**ΔG_**non-pol**_**	**Δ${\text{G}}_{\text{MM/GBSA}}^{\text{a}}$**	**${\text{IC}}_{50}^{\text{b}}$**
HDAC1–FK228	−12.47	−40.38	21.21	−5.37	−37.01	3.97
HDAC6–FK228	−8.18	−24.68	11.53	−4.51	−25.84	787

*^a^Calculated MM/GBSA binding free energies in this study*.

*^b^IC_50_ values obtained from previous study (Yurek-George et al., 2007)*.

**Figure 7:**
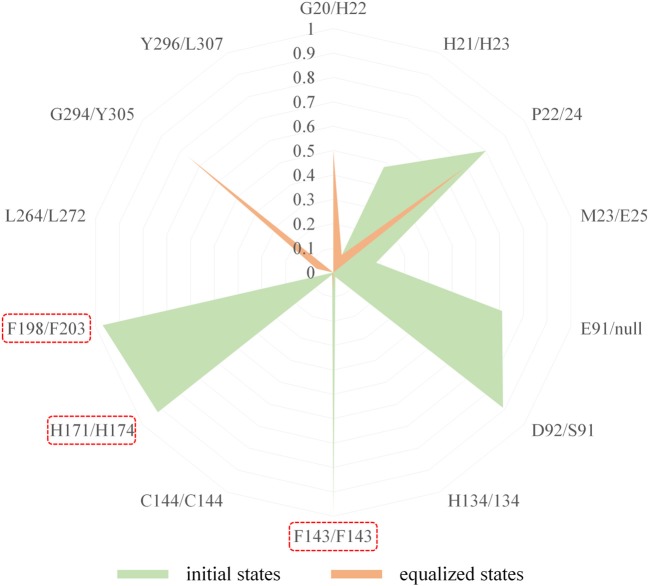
Comparison of the interaction fingerprints of FK228 in HDAC1&6 under the equilibrium trajectories.

In comparison with the residues D92 located at Loop2 of HDAC1, the reduction of the energy of corresponding residues S91 of HDAC6 contributed to FK228's binding enhanced our understanding of the difficulty of FK228 to maintain the initial conformation in HDAC6 of during MD simulation. As a result, the large spatial shift of FK228 in the binding site of HDAC6 led to the decreased energy contribution of the amino acids in the active site of HDAC6, such as F143, H174, and F203 (Figure 6), consisted very well with the decrease in the interacting frequency with these residues when compared with HDAC1 (Figure 7). For the Zn^2+^, the calculated energy contributing to FK228 binding in the active site of HDAC1 was −0.75 kcal/mol, while there was no energy contribution in HDAC6 system (Figure 6).

#### The Key Role of Residue D92 in FK228 Binding to HDAC1

**Figure 8:**
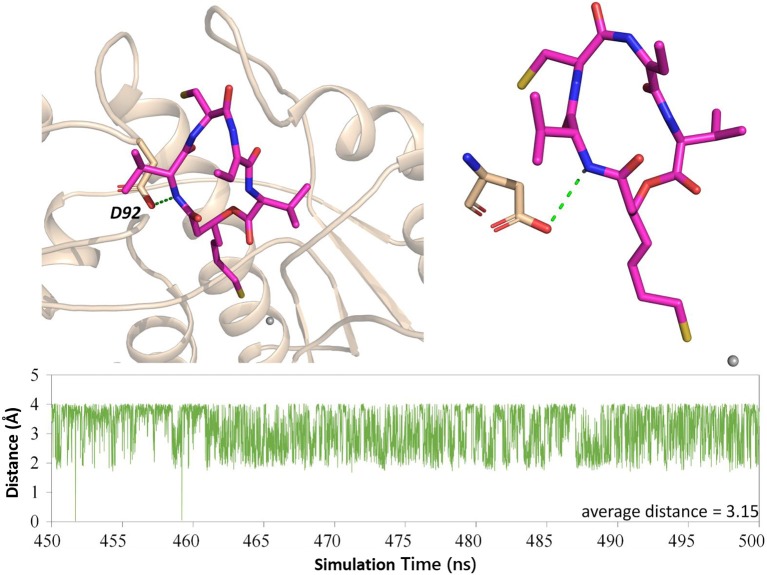
H-bond analysis between D92 and FK228 in the two constructed systems.

Both interaction fingerprints and amino acid energy contribution analysis found that residue D92 plays a key role in FK228 binding to HDAC1. The representative conformation obtained from the MD simulation trajectory in Figure 8 showed that the carbonyl group of D92 and nitrogen atom on the Cap group of FK228 could form a hydrogen bond. To further explore the huge difference in energy contribution of D92 in HDAC1 and its corresponding amino acid S91 in HDAC6, the distance between the two atoms forming the hydrogen bond during the equilibrium simulation (400–500 ns) was monitored, and the average distance between the two atoms forming hydrogen bonds was 3.15 Å (Figure 8). However, the side chain of S91 in HDAC6 lacked the hydrogen bond acceptor and its hydrophobic interaction with FK228 would gradually disappear with the deflection of its spatial position during the MD simulation (Figures 4B, 5B).

#### The Active Site Radius of Gyration Confirmed the Trend of FK228 to HDAC1

**Figure 9:**
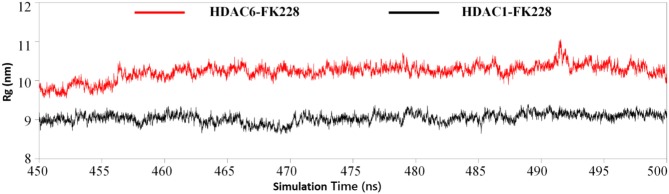
Analysis of radius of gyration of the two studied systems.

Physical and structural properties of the active pockets are closely related to the binding affinities of the ligands (Narang et al., 2019; Thillainayagam et al., 2019), the calculated binding free energy (Table 1) has successfully predicted the higher binding affinity of FK228 to HDAC1. To further evaluate the interactions between the FK228 and HDAC1&6, the radius of gyration (Rg) for the Ca-atoms of HDAC1&6 active pockets, which could be applied as an important and effective parameter to evaluate the structural integrity and compactness of the studied systems. The time evolution plot of Rg was calculated and shown in Figure 9. It is noted that the average Rg value of HDAC1-FK228 system is lower than that of HDAC6-FK228. The lower value of Rg of HDAC1-FK228 system indicated that the binding pocket of HDAC1 much more compacted and that FK228 could stay stably at the active site, which provided a guarantee for stronger interaction between FK228 and amino acids in the active pocket of HDAC1.

### Overall Comparison of the Binding Conformations of FK228 in HDAC1and6

**Figure 10:**
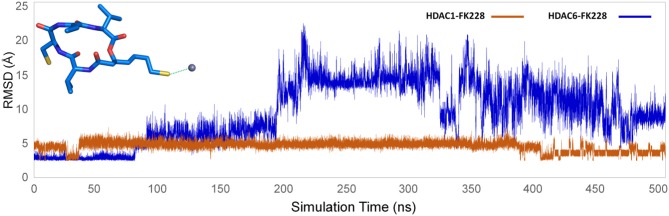
Distance between the sulfur atom in the ZBG of FK228 and zinc ion in the studied systems.

**Figure 11:**
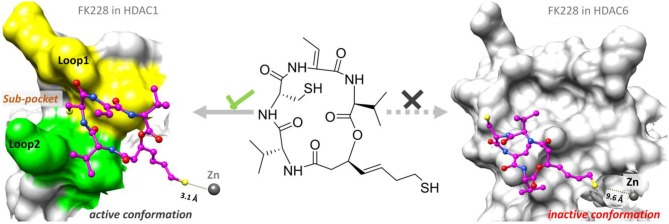
Comparison of the binding pattern of FK228 in HDAC1&6.

According to previous studies, HDAC inhibitors could exert the inhibitory activities by chelating with zinc ion at the catalytic center via the ZBG group deep into the bottom of the active pocket. According to Figure 10, the distance between the zinc ion and the sulfur atom on the ZBG of FK228 varied greatly in the two studied systems. In the HDAC1-FK228 system, the distance between the zinc ion and the sulfur atom on the ZBG of FK228 was about 3.5 Å, but the relative positions of sulfur and zinc ion is relatively larger in HDAC6-FK228 system. Furthermore, it can be learnt that Loop1 and Loop2 of HDAC1 formed a sub-pocket during the MD simulation process that could well-accommodate the sulfhydryl group on the Cap group and anchored the Cap group (Figure 11). The anchoring effects of Loop1 and Loop2 played a vital role in maintaining the binding conformation of FK228, and the relatively small spatial biases ensured the interaction of FK228 with important amino acids in the HDAC1 active pocket. However, for the HDAC6-FK228 system, the ZBG group did not penetrate the active pocket bottom to compete with the protein for metal zinc ions, and the previously calculated Rg value also indicated that the HDAC6 active pocket is less compacted, reducing the potential for interaction with FK228, which was the main reason for the large deflection of the Cap group at the active pocket.

## Conclusion

As the first approved macrocyclic HDAC inhibitor, FK228 was used as a molecular probe to compare its binding conformation in HDAC1 and HDAC6, and to explore the molecular mechanism of FK228' tendency to inhibit HDAC1 at the atomic level through a variety of *in silico* approaches. For HDAC6-FK228 system, the disappearing hydrophobic interaction of S91 (located in the HDAC6 Loop2 region) with FK228 during the MD simulation and the lack of the corresponding residue of E91 (located in the HDAC1 Loop2 region) together weakened the anchoring effects of HDAC6 Loop2 to the FK228 Cap during the MD process, leading to the large spatial conformational deviation of the docking conformation and resulting the reduced interaction between the FK228 Linker region and the conserved amino acids in the HDAC6 pocket. In the case of HDAC1-FK228 system, K228 could maintain the interactions with D92 (hydrogen bonding) and E91 (hydrophobic interaction) on Loop2 after the dynamic trajectory reached equilibrium, and the interactions with H21 and P22 on Loop1 were also strengthened. Moreover, in the process of molecular dynamics simulation, Loop1 and Loop2 on HDAC1 could form a sub-pocket that better accommodated the Cap group of FK228, maintaining the active conformation of FK228 at the binding pocket and ensuring ZBG chelating with the zinc ion and competing with the protein for the metal zinc ion, thereby exerting the inhibitory activity on HDAC1. The interaction of the Cap group with the Loop1 and Loop2 regions contributes to maintaining the active conformation of the HDACi and should be especially considered on subsequent drug design based on selective inhibition of HDAC1.

## Materials and Methods

### The Construction of the Studied Systems

The studied systems FK228-HDAC1&6 were obtained by molecular docking using the Glide (2009) software embedded in Maestro (2009) with default parameters of standard precision. The 3D structure of FK228 was drawn by *ChemBioDraw* (Dickson et al., 2014) and saved in SDfile (^*^.sdf), then processed with *LigPrep* [*OPLS-2005* (Price and Brooks, 2005) force fields] to generate the low-energy stable conformation. Additionally, the 3D structure of FK228 was preprocessed by Epik (2009) (*pH* = 7.0 ± 2.0) to generate the ionized state. After that, the protein structures of HDAC1&6 available in Protein Data Bank [PDB entry: 5ICN (Watson et al., 2016) and 5EDU (Hai and Christianson, 2016)] were processed by *Protein Preparation Wizard* (Maestro, 2009) module in Maestro (2009) to add the hydrogen atoms, assign protonation states and partial charges by *OPLS-2005* (Price and Brooks, 2005) force field, and minimize the whole protein crystal to prepare the receptor for molecular docking. The minimization process is completed when the RMSD value reached 0.30 Å. Furthermore, the spatial coordinates of largazole analog in HDAC8 were referred when defining the docking grid due to the similar binding pockets (Cole et al., 2011; Du et al., 2011; Decroos et al., 2015; Gantt et al., 2016). In molecular docking, 5,000 poses were generated during the initial phase of the docking calculation, out of which best 400 poses were chosen for energy minimization by 100 steps of conjugate gradient minimizations (the details shown in Supplementary Materials).

#### Molecular Dynamics (MD) Simulation

MD simulation was performed within AMBER16 (2016) using GPU-accelerated *PMEMD* on 16 cores of an array of two 2.6 GHz Intel Xeon E5-2650v2 processors and 4 pieces of NVIDIA Tesla K40C graphic card. *AMBER* force field *ff14SB* (Dickson et al., 2014) and Li/Merz ion parameters (Li and Merz, 2014; Li et al., 2015a,b) were used for the protein and SPC/E water. *General AMBER force field* 2 (*gaff2*) was applied to assign the parameters of FK228 in each complex, and the atom types and partial charges of FK228 could be derived on the basis of RESP calculation through *antechamber* (Wang et al., 2006). In addition, the geometrical optimization and electrostatic potential calculation for FK228 were conducted at HF/6-31G^*^ level through *Gaussian 09* software (Gaussian 09, 2009). The Zn^2+^ was processed by 12-6-4 model (Li et al., 2015a) imbedded in *Amber16*. When the constructed systems were processed by LEaP (AMBER16, 2016), it could be found that FK228-HDAC1 and FK228-HDAC6 systems were solvated with a cubic water box, and the vdw box sizes were 527706.39 Å^3^ (12,126 water molecules) and 510534.33 Å^3^ (11,910 water molecules), respectively. In addition, there were two sodium ions in HDAC1-FK228 system that were used to neutralize the negative charge, and six sodium ions were used to neutralize the negative grid charge in the HDAC6-FK228 system.

Before the MD simulation, the processed research systems were subjected to the initial energy minimization through two procedure (Xue et al., 2018a; Zhang et al., 2019). The first step was to apply harmonic restraint on solute atom (force constant = 10 kcal·mol^−1^·Å^−2^), and the second step was to release all atoms to move freely. In each step, energy minimization was conducted by the steepest descent method for the first 5,000 steps and the conjugated gradient method for the subsequent 5,000 steps. Then, each studied system was heated from 0 to 100 K and then gradually to 310 K with the protein restrained over 100 ps in the NVT ensembles. Subsequently, 10 times (5 ns) unrestrained equilibration at 310 K were performed to equilibrate system's periodic boundary condition. Finally, the unrestrained 500 ns production simulation was conducted for the prepared four systems in NPT ensembles under the temperature of 310 K and the pressure of 1 atm. Temperature was controlled by Langevin dynamics and the pressure was controlled using *Monte Carlo barostat* (2016). In all the simulations, *Particle-mesh Ewald* (PME) (Darden et al., 1993) was used to handle the long range electrostatic interaction, and *SHAKE algorithm* was exploited to keep all bonds rigid (Larini et al., 2007; Fu et al., 2018; Xue et al., 2018a; Yang et al., 2018). Time step of simulation was set 2.0 fs and a 10.0 Å cutoff was used for non-bonded interactions (Xue et al., 2018b; Du et al., 2019).

All the analysis of MD trajectories, including as root mean square deviation (RMSD), the representative structures from the trajectories, binding free energies, were analyzed and predicted via *cpptraj* and *mm_pbsa.pl* programs embedded in *AMBER16*. Structural visualization was performed in *PyMOL* software (PyMOL 1.3^1^).

### Protein-Ligand Interaction Fingerprints Analysis

Interaction fingerprints between the FK228 and the HDAC1&6 were calculated via *Ichem* (Da Silva et al., 2018; Southan, 2018), and the calculation system mainly consisted of the ligand and the binding site (residues within 6 Å of the FK228's mass center). Firstly, conformation optimization and energy optimization were carried out for the docking poses of FK228 in HDAC1&6, and then interaction fingerprints was applied to carry out for the optimized conformations to calculate the interaction between FK228 and receptors in the initial conformation. Secondly, 500 snapshots were extracted from the equalized simulated trajectories (between 400 and 500 ns) to indicate the interacting effects between the FK228 and HDAC1&6, which was compared with interactions of the initial states of the studied systems. During the process of calculation, seven important interactions (hydrophobic interaction, aromatic, H-bond donor, H-bond acceptor, positively ionizable, negatively ionizable, and metal coordination) were applied to assess the interaction fingerprints between the ligand and receptor by parsing atoms and bond connectivity fields in the form of one-dimensional (1D) descriptors consisting of 1 and 0, and the results were shown by radar plots. In addition, detailed information about the rules of detecting the interactions between protein and ligand were shown in Table S3.

### Calculation of the Binding Free Energy

MM/GBSA approach using a single molecular dynamic trajectory was adopted to calculate the binding free energy (ΔG_MM/GBSA_) regardless of entropic influence between the docked ligands and the receptor (Chen et al., 2017; Wang et al., 2017a, 2019; He et al., 2018), and in this study, 500 snapshots were extracted from the equilibrium trajectories (450–500 ns) for calculation via cpptraj. The calculation equation was as follows:

(1) $$\begin{array}{l} G_{MM/GBSA}=\Delta E_{vdW}+\Delta E_{ele}+\Delta G_{pol}+\Delta G_{nonpol} \end{array}$$

Where, Δ*E*_*vdW*_ represented the van der Waals interactions contribution, Δ*E*_*ele*_ stood for the electrostatic energy contribution, Δ*G*_*pol*_ was the polar solvent interaction energy calculated with the GB model (*igb* = 2) and *G*_*nonpol*_ was the non-polar solvation free energy, which was evaluated using LCPO method (0.0072 × ΔSASA, SASA indicating the solvent accessible area with a probe radius of 1.4 Å) (Weiser et al., 1999; Zheng et al., 2016).

### Calculating the Per-Residue Energy Contribution

The per-residue energy contribution $\Delta G_{MM/GBSA}^{per-residue}$ between the residues located in HDAC1&6 and the docked ligands was calculated using the following formula:

(2) $$\begin{array}{l} \Delta G_{MM/GBSA}^{per-residue}=\Delta E_{vdW}^{per-residue}+\Delta E_{ele}^{per-residue}\\ \;+\Delta G_{pol}^{per-residue}+\Delta G_{nonpol}^{per-residue} \end{array}$$

Where, the three terms, namely ($\Delta E_{vdW}^{per-residue}$), $\Delta E_{ele}^{per-residue}$ and $\Delta G_{pol}^{per-residue}$, were defined in the same way as the corresponding terms in formula 2, and $\Delta G_{nonpol}^{per-residue}$ was calculated using the *ICOSA* method (Wang et al., 2006, 2017b).

### Radius of Gyration Calculation

The residues consisting of the binding site were selected to calculate the radius of gyration of the studied systems, and there were 30 residues in each systems. In this study, the equilibrium trajectories (450–500 ns) were used to calculate the Rg of the specified residues via *cpptraj*.

## Data Availability Statement

All datasets generated for this study are included in the article/Supplementary Material.

## Author Contributions

FZ and WX conceived the work and directed the experiments. YZ performed the MD simulations. YZ, TF, YR, FL, GZ, JH, and XY collected and confirmed the data of protein and ligand structures and performed the analysis. FZ, WX, and YZ drafted the first and second version of the manuscript. All authors read, edited, and approved the final version of manuscript.

### Conflict of Interest

The authors declare that the research was conducted in the absence of any commercial or financial relationships that could be construed as a potential conflict of interest.

## Footnotes

^1^PyMOL Molecular Graphics System, *v*. 1.3. *Schrödinger*, LLC, New York, NY.

## Abbreviations

Cap: capping group
CD: catalytic domain
CU: connect unit
GBM: Glioblastoma
HATs: histone acetyltransferases
HDAC6: Histone deacetylase 6
HDACi: HDAC inhibitors
MD: Molecular Dynamics
MM/GBSA: Molecular Mechanics Generalized Born Surface Area
sHDAC6Is: selectivity of HDAC6 inhibitors
ZBG: zinc binding group
