# Supplementary material for: Selective Inhibition of HDAC1 by Macrocyclic Polypeptide for the Treatment of Glioblastoma: A Binding Mechanistic Analysis Based on Molecular Dynamics
Source: Front Mol Biosci. 2020 Mar 11;7:41. doi: 10.3389/fmolb.2020.00041 (PMC7078330; doi:10.3389/fmolb.2020.00041)
Supplement: Supplementary file 1 [file Data_Sheet_1.pdf]

Supplementary Materials for:

Selective Inhibition of HDAC1 by Macrocyclic Polypeptide for the Treatment of Glioblastoma: A Binding Mechanistic Analysis Based on Molecular Dynamics

Yang ZHANG[§,†], Tingting FU[†], Yuxiang Ren[§], Fengcheng LI[§], Guoxun ZHENG[†], Jiajun HONG[§], Xiaojun YAO[‡], Weiwei XUE[†,*] and Feng ZHU[§,†,*]

[§] College of Pharmaceutical Sciences, Zhejiang University, Hangzhou 310058, China

[†] School of Pharmaceutical Sciences, Chongqing University, Chongqing 401331, China

[‡] State Key Laboratory of Applied Organic Chemistry and Department of Chemistry, Lanzhou University, Lanzhou 730000, China

* Corresponding Author: Prof. Feng ZHU, College of Pharmaceutical Sciences, Zhejiang University, Hangzhou 310058, China. Email: zhufeng@zju.edu.cn; prof.zhufeng@gmail.com. Asso/Prof. Wei Wei XUE, School of Pharmaceutical Sciences, Chongqing University, Chongqing 401331, China. Email: xueww@cqu.edu.cn.

Supplementary materials for

MATERIALS AND METHODS

The Construction of the Studied Systems

In this study, *Glide* with standard precision was applied for molecular docking (keeping the prepared receptors rigid, and keeping docked ligands flexible, namely sampling nitrogen inversions and sampling ring conformations). 400 and 5000 are the parameters of "*Output*" (specify the type of file to create the output ligand poses and to determine how many poses to write, per ligand and per docking job), and the specific parameters were set as follows: (1) Write out at most 5000 ligand poses per docking run; (2) Write out at most 400 poses per ligand, which were than subject to post-docking minimization. In addition, the per-residue interaction scores for residues within 12.0 Å, and the RMSD values to the input ligand geometries were also applied during the calculation process.

SI Tables

Table S1. Detailed information of the initial conformations of FK228 in HDAC1.

Entry ID	RMSD [a]	Docking Score [b]
**1** (*first simulation*)	**0.436**	**-6.631**
**2** (*second simulation*)	**0.482**	**-5.563**
3	0.662	-5.510
4	0.797	-5.387
5	0.711	-5.317
6	0.716	-5.154
7	0.897	-4.747
8	1.008	-4.629
9	1.008	-4.506
10	1.018	-4.483
11	1.018	-4.478
12	1.068	-4.366
13	1.268	-4.353
14	1.300	-4.135
15	1.359	-4.087
16	1.358	-4.010
17	1.356	-3.917
18	1.387	-3.910
19	1.388	-3.904
20	1.341	-3.897
21	1.343	-3.891
22	1.388	-3.825
23	1.610	-3.808
24	1.615	-3.777
25	1.615	-3.774
26	1.617	-3.763
27	1.823	-3.720
28	1.856	-3.650
29	1.911	-3.615
30	1.973	-3.607
31	1.985	-3.595
32	1.885	-3.562
33	1.866	-3.554
34	1.977	-3.549
35	2.033	-3.530
36	2.037	-3.523
37	2.039	-3.503
38	2.102	-3.491
39	2.933	-3.490

40	3.066	-3.484
41	2.672	-3.470
42	2.682	-3.462
43	2.687	-3.407
44	2.682	-3.395
45	2.677	-3.382
46	3.132	-3.374
47	3.367	-3.328
48	3.292	-3.246
49	3.662	-3.233
50	3.692	-3.221
51	3.786	-3.152
52	3.779	-3.199
53	3.677	-3.165
54	3.682	-3.123
55	3.615	-3.109
56	3.790	-3.098
57	3.680	-3.085
58	3.329	-3.077
59	3.683	-3.072
60	3.632	-3.065
61	3.678	-3.057
62	3.678	-3.032
63	3.668	-3.026
64	3.610	-3.015
65	3.642	-3.010
66	3.621	-3.009
67	3.692	-3.007
68	3.677	-2.992
69	3.628	-2.991
70	3.645	-2.974
71	3.643	-2.961
72	3.396	-2.958
73	3.587	-2.929
74	3.217	-2.886
75	3.982	-2.876

[a] RMSD between the docked pose and the original ligand in the crystal

[b] Docking score provide by *Glide*

Table S2. Detailed information of the initial conformations of FK228 in HDAC6.

Entry ID	RMSD [a]	Docking Score [b]
**1** (*first simulation*)	**0.495**	**-5.783**

**2** (*second simulation*)	**0.515**	**-5.182**
3	0.765	-5.173
4	0.766	-5.171
5	0.765	-5.154
6	0.766	-5.128
7	0.767	-5.117
8	0.882	-4.988
9	1.182	-4.932
10	1.587	-4.867
11	1.688	-4.804
12	1.820	-4.797
13	1.189	-4.785
14	2.002	-4.680
15	1.987	-4.673
16	1.889	-4.593
17	2.112	-4.550
18	2.209	-4.527
19	2.068	-4.471
20	2.677	-4.418
21	2.689	-4.383
22	2.782	-4.379
23	2.627	-4.377
24	2.052	-4.345
25	3.282	-4.306
26	2.912	-4.280
27	2.687	-4.250
28	3.212	-4.239
29	2.778	-4.222
30	2.772	-4.212
31	2.693	-4.199
32	2.987	-4.196
33	3.225	-4.168
34	3.620	-4.096
35	3.627	-4.067
36	3.688	-3.999
37	3.483	-3.983
38	3.440	-3.972
39	3.563	-3.929
40	3.566	-3.918
41	3.511	-3.886
42	3.677	-3.881
43	3.622	-3.817
44	3.701	-3.816

45	3.682	-3.808
46	3.688	-3.709
47	3.687	-3.688
48	3.682	-3.587
49	3.679	-3.571
50	3.683	-3.569
51	3.678	-3.566
52	3.679	-3.545
53	3.678	-3.517
54	3.668	-3.508
55	3.679	-3.498
56	3.678	-3.477
57	3.986	-3.398
58	3.678	-3.376
59	3.990	-3.298
60	3.619	-3.268
61	3.782	-3.174
62	3.682	-3.122
63	3.683	-3.082
64	3.783	-3.001
65	3.799	-2.998
66	3.985	-2.618
67	3.987	-2.556
68	3.985	-2.238

[a] RMSD between the docked pose and the original ligand in the crystal

[b] Docking score provide by *Glide*

Table S3. The rules of detecting protein-ligand interactions[1].

**Interaction type**	**Protein atoms**	**Ligand atoms**	**Rule 1**[a]	**Rule 1**[b]
Hydrophobic	Hydrophobic	Hydrophobic	$\lVert\overrightarrow{Y_1Y_2}\rVert \leq 4.5$ Å	
Aromatic (face to face)	Aromatic	Aromatic	$\lVert\overrightarrow{\alpha_1\alpha_2}\rVert \leq 4$ Å & $\lVert\overrightarrow{\alpha_i\alpha_j}\rVert \leq 12$ Å	$\langle\overrightarrow{n_1},\overrightarrow{n_2}\rangle \in \left[\frac{-\pi}{6},\frac{\pi}{6}\right]$
Aromatic (edge to face)	Aromatic cycle	Aromatic cycle	$\lVert\overrightarrow{\alpha_1\alpha_2}\rVert \leq 4.0$ Å[c]	$\langle\overrightarrow{n_1},\overrightarrow{n_2}\rangle \in \left[\frac{\pi}{6},\frac{5\pi}{6}\right]$
H-bond (Protein: acceptor)	H-bond acceptor	H-bond donor	$\lVert\overrightarrow{DA}\rVert \leq 3.5$ Å	$\langle\overrightarrow{DH},\overrightarrow{HA}\rangle \in \left[\frac{-\pi}{4},\frac{\pi}{4}\right]$
H-bond (Protein: donor)	H-bond donor	H-bond acceptor	$\lVert\overrightarrow{DA}\rVert \leq 3.5$ Å	$\langle\overrightarrow{DH},\overrightarrow{HA}\rangle \in \left[\frac{-\pi}{4},\frac{\pi}{4}\right]$
Ionic (Protein: anionic)	Negative ionizable	Positive ionizable	$\lVert\overrightarrow{+-}\rVert \leq 4.0$ Å	
Ionic (Protein: cationic)	Positive ionizable	Negative ionizable	$\lVert\overrightarrow{+-}\rVert \leq 4.0$ Å	

[a]Y, hydrophobe; a_1, protein interacting atom; a_2, ligand interacting atom; a_i, any atom of the protein aromatic ring; a_j, any atom of ligand aromatic ring; D, H-bond donor; A, H-bond acceptor; +, cation; -, anion; [b]n, normal to the aromatic ring; H, hydrogen. [c] for 5 pairs of protein-ligand interacting aromatic atoms.

SI Figures

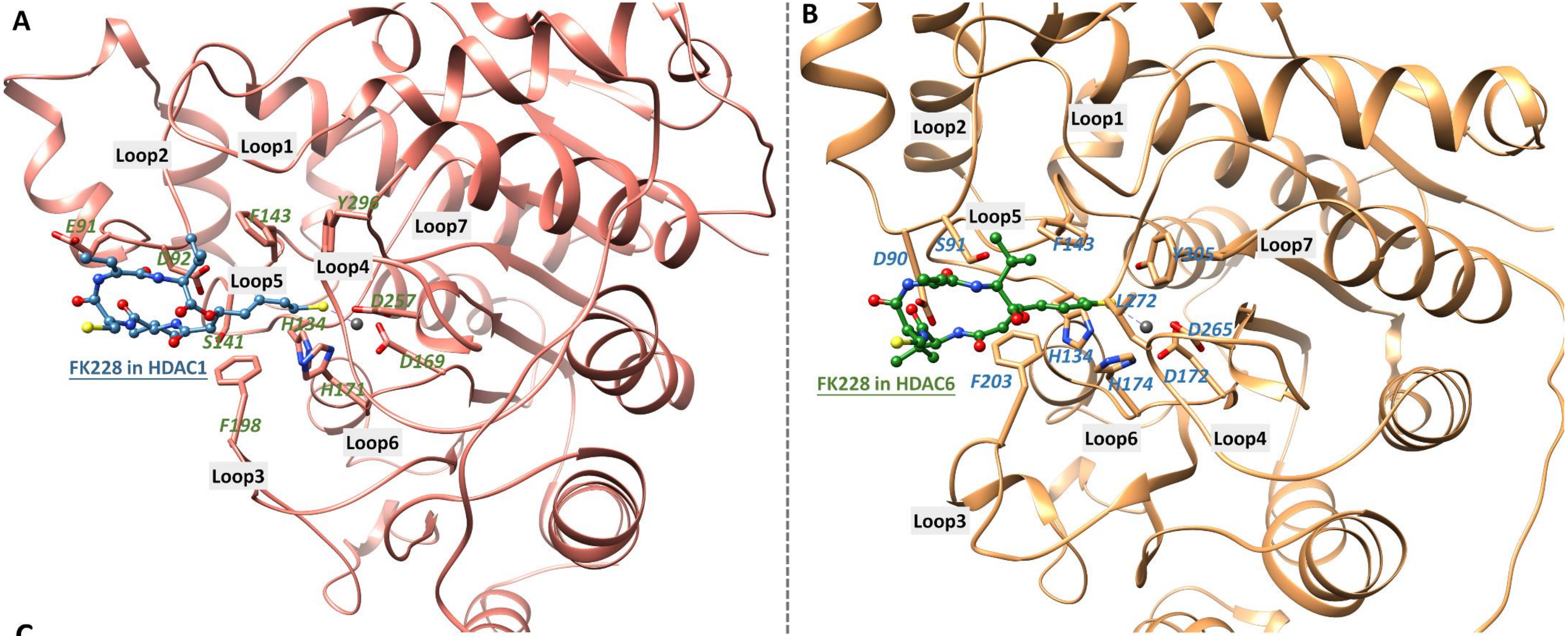

C

HDAC1	G20	H21	P22	M23	K24	H26	E91	D92	C93	H133	H134	A139	S141	G142	F143	C144	D169	I170	H171	Y197	F198	P199	D257	G261	D262	R263	L264	G294	G295	Y296	T297	N300	Zn
HDAC6	H22	H23	P24	E25	V26	Q28	Null	S91	I92	H133	H134	A139	C141	G142	F143	C144	D169	V173	H174	F202	F203	P204	D265	G269	D270	P271	L272	Y305	N306	L307	T308	S309	Zn
HDAC8	K20	I21	P22	K23	R24	Null	Y87	D88	C89	H129	H130	A136	S137	G138	F139	C140	D165	L166	H167	F194	F195	P196	D254	G258	D259	P260	M261	G291	G292	Y293	N294	N297	Zn

Figure S1. Global comparison of the binding sites on HDAC1 and HDAC6: (A) binding conformation of FK228 in HDAC1; (B) binding conformation of FK228 in HDAC6, and the non-conserved amino acids were marked in red; (C) sequence alignment of the residues mainly located in loop 1-7 of HDAC1, 6, and 8 (nonconserved amino acids were marked in red).

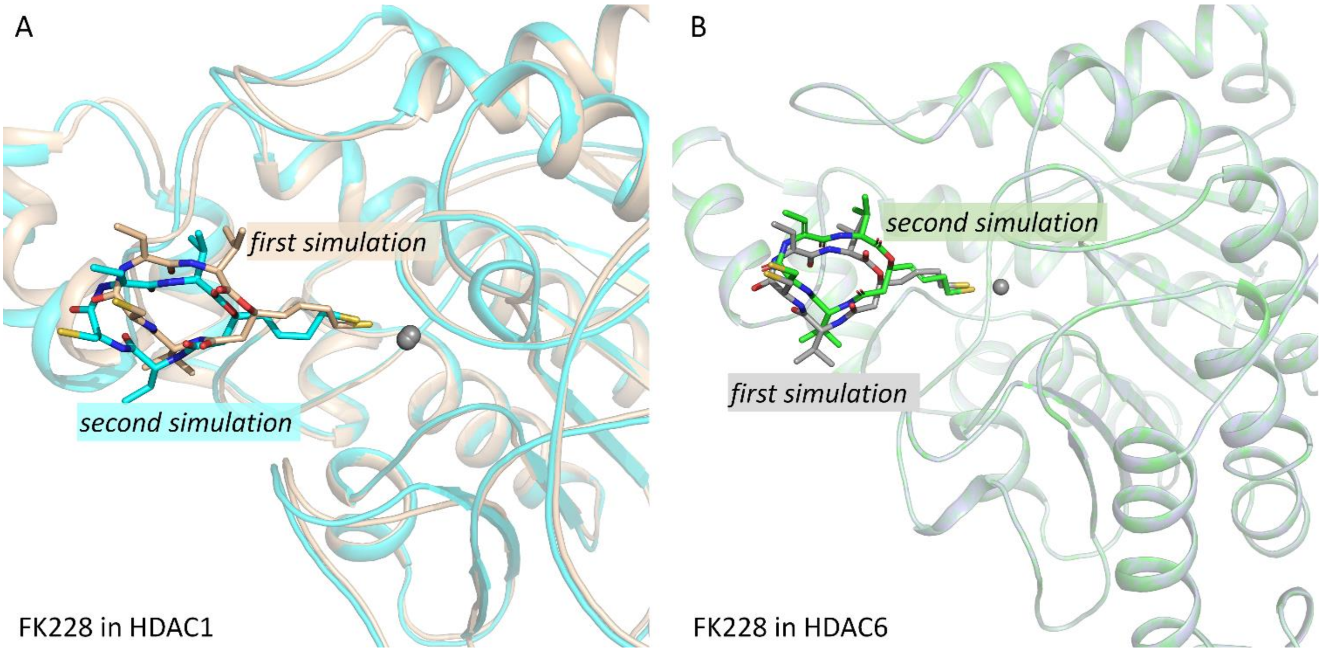

Figure S2. The initial conformations of FK228 in HDAC1&6 of the two simulations.

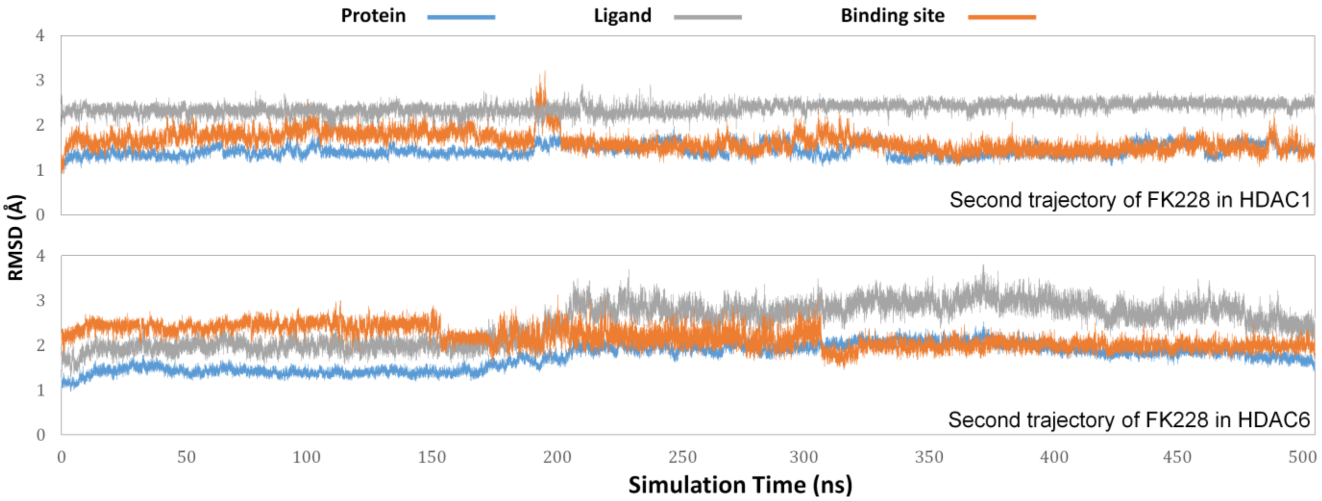

Figure S3. Root mean square deviations of protein backbone atom, ligand heavy atoms, and the backbone atoms of the residues in the binding site as the function of time in MD simulations of the additional independent experiments.

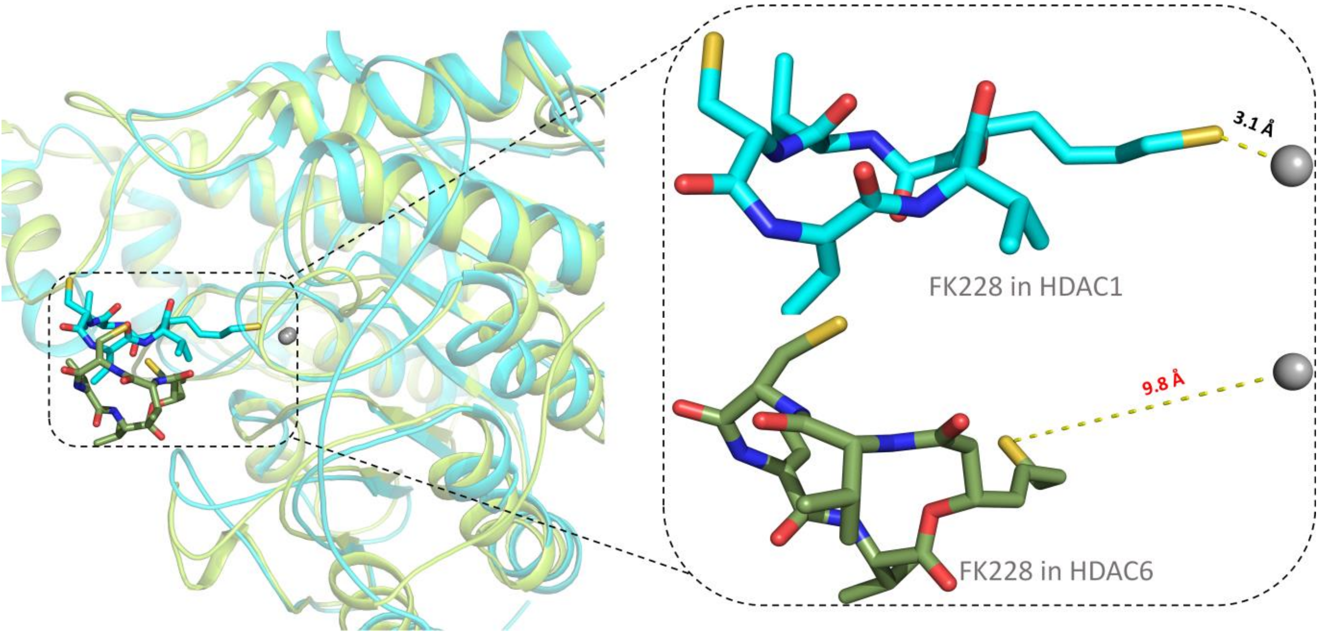

Figure S4. Comparison of the representative conformation of FK228 in HDAC1&6 of the additional independent experiments.

Reference

1. J. Desaphy, E. Raimbaud, P. Ducrot and D. Rognan, *J. Chem. Inf. Model.*, 2013, **53**, 623-637.
